# Intramedullary spinal capillary hemangioma with secondary neurulation defect in children

**DOI:** 10.1007/s00381-024-06276-0

**Published:** 2024-01-19

**Authors:** Jong Seok Lee, Ji Yeoun Lee, Sung-Hye Park, Kyu-Chang Wang, Kyung Hyun Kim

**Affiliations:** 1 Division of Pediatric Neurosurgery, Seoul National University Children’s Hospital, Seoul, Republic of Korea; 2 Department of Neurosurgery, Seoul National University College of Medicine, Seoul, Republic of Korea; 3 Neural Development and Anomaly Laboratory, Department of Anatomy and Cell Biology, Seoul National University College of Medicine, Seoul, Republic of Korea; 4 Department of Pathology, Seoul National University College of Medicine, Seoul, Republic of Korea; 5 Neuro-Oncology Clinic, Center for Rare Cancers, National Cancer Center, Goyang, Republic of Korea

**Keywords:** Spinal cord neoplasm, Vascular malformation, Capillary hemangioma, Spinal dysraphism, Tethered cord syndrome

## Abstract

Intramedullary spinal capillary hemangioma is a rare occurrence in pediatric patients, and only limited cases have been reported. This study presents the first two cases of spinal capillary hemangioma co-present with retained medullary cord and one case of spinal capillary hemangioma with lumbosacral lipomatous malformation. Previous literature on ten patients with this pathology was reviewed. We speculated pathogenesis, imaging features, and histopathologic findings of the disease.

## Introduction

Capillary hemangioma is a benign hamartoma resulting from the proliferation of vascular endothelial cells. It usually arises in the skin or mucosa of the head and neck [1–3]. Spinal capillary hemangioma is very rare. It can arise anywhere in the spinal cord, extradural, intradural and extramedullary, or intramedullary space [1, 3, 4]. It is considered a congenital lesion, but most previous reports included adult patients [1, 5]. To the best of our knowledge, only nine reports on pediatric intramedullary spinal capillary hemangioma have been documented [2–4, 6–11]. Recently, we encountered two infants with this pathology who were co-present with retained medullary cord (RMC), and reviewed one patient with lumbosacral lipomatous malformation (LLM). Based on the clinical findings and review of previous literature, we described the clinical features, pathophysiology, and precautions for diagnosis.

## Case description

### Patient 1

**Fig. 1:**
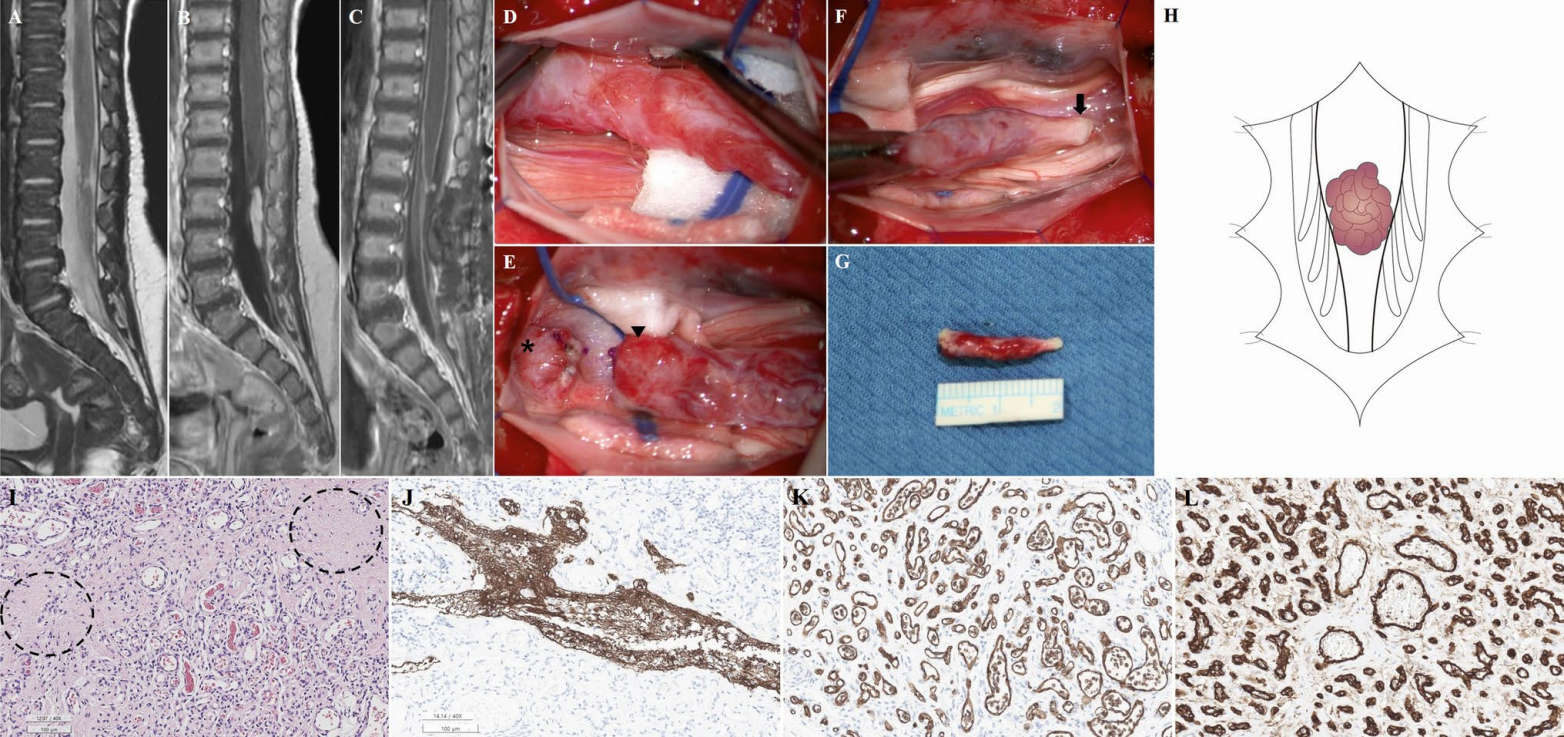
Magnetic resonance imaging (MRI, **A**–**C**), intraoperative images (**D**–**G**), schematic illustration (**H**), and histopathologic findings (**I**–**L**) of RMC with capillary hemangioma in patient 1. **A** T2-weighted sagittal MRI and **B**, **C** contrast-enhanced sagittal MRI. **A**, **B** There is a 0.4 × 0.5 × 1.9 cm-sized, T2-isointense and contrast-enhanced mass in the distal conus medullaris and the filum terminale, corresponding L3-4 spinal level. **C** Postoperative MRI shows no residual lesion. **D** Thick and tortuous vessels encase the surface of RMC and filum terminale, which show no response to intraoperative electrical stimulation. **E** The non-functioning cord (arrowhead) is amputated above the tortuous vessels. The distal functioning cord (asterisk) is preserved. **F** The filum terminale (arrow) is amputated below the tortuous vessels. **G** The resected RMC and filum terminale containing tortuous vessels are biopsied for histopathologic evaluation. **H** The illustration shows a typical RMC with capillary hemangioma. **I** Photomicrographs showing capillary vessels intermixed with glial tissue (dotted circles). The capillaries are lined by endothelial cells, and some of them are filled with red blood cells. (Hematoxylin and eosin (H&E) stain, Underbar scale: 100 µm). (J) GFAP is positive in the glial tissue among the hemangioma. **K**, **L** The capillary endothelial cells are positive for GLUT1 and CD31 (**I**, H&E; **J**, GFAP; **K**, GLUT-1; **L**, CD31)

A 3-month-old female presented a deviated gluteal fold and perianal cutaneous hemangioma without neurological deficits. The spinal magnetic resonance imaging (MRI) showed a 0.4 × 0.5 × 1.9 cm-sized, T2-isointense and contrast-enhanced mass in the conus medullaris and filum terminale, corresponding L3-4 level (Fig. 1A, B). Radiological diagnoses were filar fibrolipoma or intradural extramedullary tumors such as myxopapillary ependymoma or schwannoma. There showed left L5-S1 radiculopathy with partial axonotmesis in electromyography (EMG) and nerve conduction study (NCS), and acontractile detrusor with low compliance in the urodynamic study (UDS). Because the lesion could be a tumor or it could cause a tethered cord, surgical treatment was decided.

L2-3 laminotomy was performed. Upon opening the dura, thick and tortuous vessels encased the surface of the distal conus medullaris and the proximal filum terminale (Fig. 1D). The distal portion of the cord showed no response to intraoperative electrical stimulation, which corresponded to RMC. The distal non-functioning cord was amputated above the tortuous vessels, and the functioning cord was preserved (Fig. 1E). The filum terminale was amputated below the tortuous vessels (Fig. 1F). The resected RMC containing tortuous vessels were biopsied for histopathologic evaluation (Fig. 1G).

The histopathologic findings showed central glioependymal tissue and infantile capillary hemangioma (Fig. 1I–L). Immunohistochemically, the glioependymal tissue was positive for glial fibrillary acidic protein (GFAP), and endothelial cells were positive for glucose transporter 1 (GLUT1) and the cluster of differentiation 31 (CD31), which corresponded to juvenile capillary hemangioma. Postoperative MRI showed no residual lesions (Fig. 1C). The patient showed no deficit at the 3-month follow-up.

### Patient 2

**Fig. 2:**
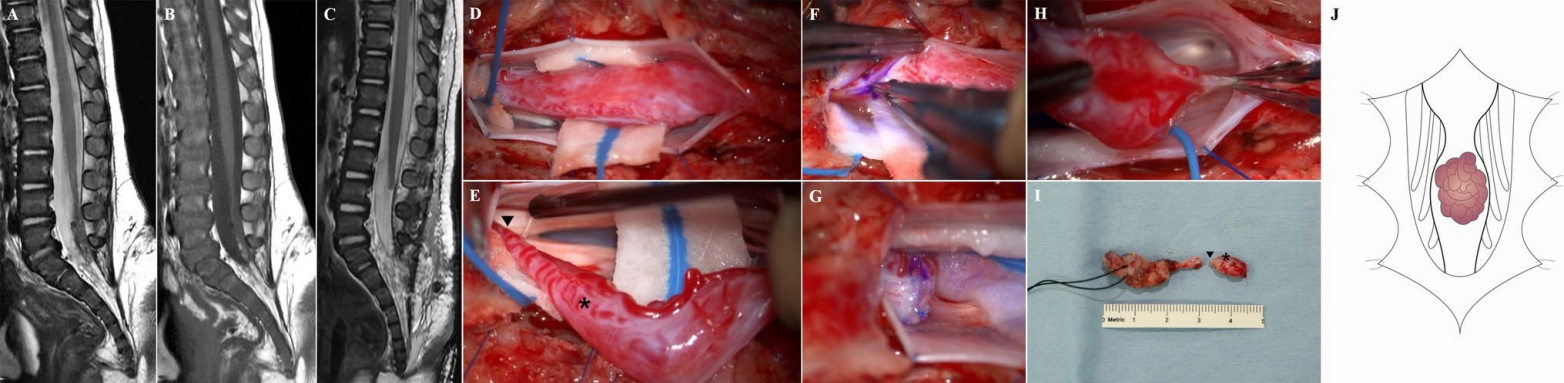
Magnetic resonance imaging (MRI, **A**–**C**), intraoperative images (**D**–**I**), and schematic illustration (**J**) of RMC with capillary hemangioma in patient 2. **A**, **C** T2-weighted sagittal MRI and **B** T1-weighted sagittal MRI. **A**, **B** The spinal cord narrows to form a thick filum-like structure and widens again, forming an hourglass shape. There is a T1- and T2-hypointense thread-like structure on the surface of the cord (filum-like structure and rewidened cord) at the L5-S1 spinal level. The intradural fibrous stalk with small fatty tissue beginning at the S1 spinal level exits through the posterior spinal defect at the S3 spinal level and extends to the subcutaneous layer. **C** Postoperative MRI shows no residual lesion. **D** Upon opening the dura, the LDM stalk is attached to the dorsal surface of the cord at the S1 spinal level. **E** Thick and tortuous vessels encase the filum-like structure (arrowhead) and rewidened cord (asterisk). They show no response to intraoperative electrical stimulation, which corresponds to RMC. **F**, **G** The non-functioning filum-like structure is amputated above the tortuous vessels. **H** The distal portion of the rewidened cord (RMC) is amputated below the tortuous vessels, and the LDM stalk is resected. **I** The resected LDM stalk and RMC (arrowhead and asterisk) containing tortuous vessels are biopsied for histopathologic evaluation. **J** The illustration shows that capillary hemangioma encases the filum-like structure and RMC

A 2-month-old female presented a sacral cutaneous hemangioma and “cigarette-burn” skin lesion without neurological deficits. The spinal MRI showed low-lying conus (Fig. 2A, B). The spinal cord narrowed to form a thick filum-like structure and widened again, forming an hourglass shape. There was a T1- and T2-hypointense thread-like structure on the surface of the filum-like structure and rewidened cord at the L5-S1 level. Also, the intradural stalk with small fatty tissue beginning at the S1 level exited through the posterior spinal defect at the S3 level and extended to the subcutaneous layer, which was compatible with limited dorsal myeloschisis (LDM). EMG/NCS revealed the presence of left lumbosacral polyneuropathy at and below the S1 level with mild partial axonal involvement. UDS showed decreased bladder compliance, detrusor under-activity, and detrusor sphincter dyssynergia. Both findings suggested tethered cord, and surgical treatment was decided.

The fibrous tract was traced from the subcutaneous layer, and the stalk was found to be connected to intradural space. Upon opening the dura, the stalk was attached to the dorsal surface of the distal cord at the S1 level (Fig. 2D). Thick and tortuous vessels encased the filum-like structure and rewidened cord (Fig. 2E). They showed no response to intraoperative electrical stimulation, which corresponded to RMC. The non-functioning filum-like structure was amputated above the tortuous vessels (Fig. 2F, G). The distal portion of the rewidened cord (RMC) was amputated below the tortuous vessels, and the LDM stalk was resected (Fig. 2H). The resected LDM stalk and RMC containing tortuous vessels were biopsied for histopathologic evaluation (Fig. 2I).

The lesions were histopathologically diagnosed as RMC with capillary hemangioma and LDM. The patient presented no deficits without residual lesions on postoperative MRI (Fig. 2C).

### Patient 3

**Fig. 3:**
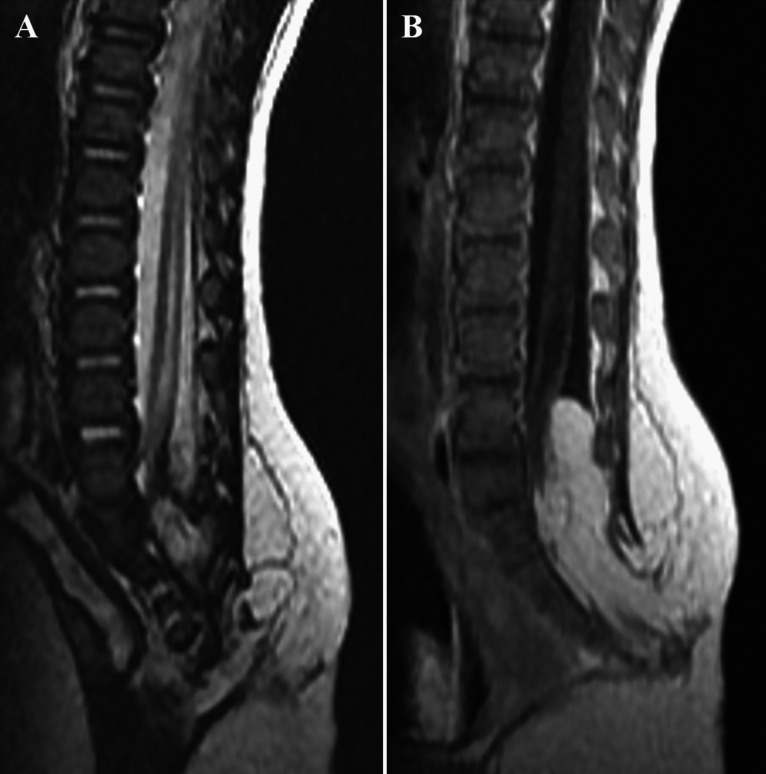
Magnetic resonance imaging (MRI) of patient 3. **A** T2-weighted sagittal MRI and **B** contrast-enhanced sagittal MRI. **A**, **B** It shows lumbosacral lipomatous malformation from L5 to S3 levels with diffuse syrinx

A 2-month-old female presented a low sacral dimple and subcutaneous mass without neurological deficits. The spinal MRI showed LLM from L5 to S3 levels with diffuse syrinx at lumbar levels (Fig. 3). There was no enhancing lesion on MRI. EMG/NCS revealed no abnormalities, but voiding cystourethrography showed grade 2 vesicoureteral reflux on the right side. Debulking of LLM and complete untethering were performed. During the operation, no prominent vascular malformation was identified. The resected lesion was histopathologically reviewed, and the diagnoses were lipoma and fistulous tract with capillary hemangioma. The patient presented no neurological deficits after the operation.

## Discussion

### Review of previous reports on pediatric spinal capillary hemangioma

Nine previous reports described ten patients with pediatric spinal capillary hemangioma [2–4, 6–11] (Table 1). Including our three cases, there were six males and seven females. Ages ranged from 1 month to 18 years. Presenting symptoms were back pain, leg pain, lower extremity weakness, and urinary difficulty. Symptoms in previous reports were caused by mass effect, and symptoms in our cases were caused by tethering of the cord. Lesions were mostly located in lumbar levels in nine patients. Among seven patients with skin stigmata, six patients showed associated spinal dysraphism such as fatty filum terminale, LLM, or dermal sinus tract. Among five patients with cutaneous hemangioma, four patients (80%) showed associated spinal dysraphism. Telangiectatic hemangiomas occurring in the lumbosacral region should be considered potential cutaneous stigmata of spinal dysraphism and, as such, warrant screening with MRI. The first-line treatment of cutaneous hemangioma is propranolol, which promotes vasoconstriction, reducing blood flow within the lesion, and inhibits angiogenesis by decreasing the expression of proangiogenic growth factors [12, 13]. Also, there was a report that showed the effectiveness of the medication for the intracranial lesion [12].

**Table 1:** Summary of ten pediatric patients with spinal capillary hemangioma in the literature and three patients in the present study

**Authors and years**	**Age/sex**	**Location**	**Conus level**	**Syrinx**	**Skin stigmata**	**Associate anomalies**	**Preoperative symptoms**	Duration of symptoms*	**Operation**	**Extent of resection**	**Postoperative symptoms**	**Recurrence (repeated surgery)**	**Follow-up periods**
**Mawk et al. (1987) **[9]	1 m/M	L2/ -	NA	No	Sacral cutaneous hemangioma at birth	No	Apraxia of legs	Short-term	Yes	STR	Normalized	No (spontaneous involution)	1 m
**Iannelli et al. (2005) **[7]	3 m/M	T4-7 IM	NA	No	No	Hydrocephalus	Irritability and lethargy	1 w	Yes (+ VP shunt)	GTR	Normalized	No	1 y
**Karikari et al. (2007) **[8]	6 m/F	L4 ED	L2-3	No	Lumbar dimple, cutaneous hemangioma	Dermal sinus tract, thickened fatty filum terminale	No	No	Yes (+ filum resection)	GTR	No	No	4 m
	1 m/F	L3-4 IDEM	L3-4	No	Lumbar cutaneous hemangioma	Dermal sinus tract, fatty filum terminale	No	No	Yes (+ filum resection	GTR	No	No	3 m
**Ganapathy et al. (2008) **[6]	17 y/M	L2-3 IDEM	L2	No	No	No	LBP, Lt. S1 radiculopathy	4 m	Yes	GTR	NA	NA	NA
**Wu et al. (2013) **[3]	18 y/M	T7-8 IM	NA	Yes	No	No	LBP, bilat. LEx. weakness, dysuria	5 m	Yes	GTR	Normalized	No	8 y 6 m
**Gencipinar et al. (2014) **[2]	17 m/F	T3-7 ED	NA	No	No	No	Bilat. LEx. weakness (unable to walk)	1 d	Yes	GTR	Improved (able to walk)	No	NA
**Tunthanathip et al. (2017) **[4]	15 y/M	L1 IDEM	L1	No	No	No	Lt. leg pain	4 m	Yes	GTR	Pain improved, transient urinary retention	No	3 m
**Naruke et al. (2019) **[10]	6 m/F	L2 IDEM	L2	No	Sacral skin tag	Fatty filum terminale	No	No	Yes	GTR	No	No	8 m
**Vaisha et al. (2019) **[11]	2 m/M	T10-12 IDEM	L2	No	No	Hydrocephalus	Increased tone and flexor spasm in bilat. LEx	NA	Yes (+ VP shunt)	GTR	Minor limp, self gait	No	2 y
**Current cases**	3 m/F	L3-4 IDEM	L3-4	No	Deviated gluteal fold, cutaneous hemangioma	Retained medullary cord	No	No	Yes	GTR	No	No	3 m
	2 m/F	L5-S1 IDEM	L4	No	Cutaneous hemangioma, cigarette burn scar	Retained medullary cord, Limited dorsal myeloschisis	No	No	Yes	GTR	No	No	1 m
	2 m/F	Unidentified on MRI	S1	Yes	Sacral dimple. Subcutaneous mass	Lumbosacral lipomatous malformation	No	No	Yes	GTR	No	No	14 y 6 m

*Bilat* bilateral, *d* day(s), *ED* epidural,* F* female, *GTR* gross total resection, *IDEM* intradural extramedullary, *IM* intramedullary, *LBP* low back pain, *LEx* lower extremity, *Lt* left, *M* male, *m* month(s), *MRI* magnetic resonance imaging, *NA* not available, *Rt* right, S*TR* subtotal resection, *VP* ventriculoperitoneal, *w* week(s), *y* year(s)

On the other hand, RMC and LDM were only presented in our patients. In patient 1, it shows a typical RMC with capillary hemangioma (Fig. 1H). In patient 2, it shows an hourglass-shaped RMC with an intervening filum-like structure between the conus and RMC. Capillary hemangioma encases the filum-like structure and RMC (Fig. 2J). This type of RMC was described in our previous literature [14]. In the second case described by Karikari et al. [8], the patient showed L3-4 intradural mass with low-lying conus. There is a possibility that the lesion could be a “possible RMC [14]”, but the functionality of the distal cord was not evaluated.

### Pathogenesis

The pathogenesis of spinal capillary hemangioma is not established well. Capillary hemangioma is usually considered to arise from hamartomatous proliferations of vascular endothelial cells, which can be found throughout the entire body [7]. However, lesions that arise in the spinal cord are considered unique, and other pathogenesis has been suggested. Especially there have been similar cases of spinal capillary hemangioma that were located in the lumbar region [4, 6, 8–10]. Some of them were co-present with spinal dysraphism, such as fatty filum terminale or dermal sinus tract [8, 10]. From the embryological viewpoint, there have been suggestions that these lesions originated from cells involved in secondary neurulation [8, 10].

During secondary neurulation, pluripotent cells of the caudal eminence give rise to the spinal cord below the S1—2 level, which starts at 26–27 days of gestation [10, 14]. The medullary cord is formed from the gathering of primitive neural stem cells of the caudal cell mass, and extensive apoptosis of the coccygeal segments of the medullary cord results in the filum terminale, which starts at 28–32 days of gestation [10, 14]. RMC is considered to be caused by complete or partial arrest in this process of secondary neurulation [14].

In a previous report of spinal capillary hemangioma associated with fatty filum terminale, which resulted from the failure of the late phase of secondary neurulation, the author suggested that lesions originated from the pluripotent cells of the caudal eminence [10]. There have been reported that some spinal lipomas contain ectopic tissues such as muscle, bone, and cartilage, and these ectopic tissues are considered to originate from the pluripotent tissues of the caudal eminence. The author suggested that the origin of spinal capillary hemangioma might be the same as that of ectopic tissues in spinal lipomas. We present the first cases of capillary hemangioma co-present with RMC. These cases also support the previous speculation that the pluripotent cell mass of “caudal eminence,” which forms the medullary cord during normal development, may also give rise to a “malformed” medullary cord containing capillary hemangioma. Similar to how RMC is formed, the present cases also may have resulted from the failure of degeneration of the medullary cord. More cases of spinal capillary hemangioma involved in spinal dysraphism should be presented to speculate more precise pathoembryogenesis of the disease.

### Radiologic differential diagnostic features and histopathologic findings

It is difficult to primarily diagnose spinal capillary hemangioma on MRI because the disease is rare, and the distinction from other spinal masses can be confusing. MRI features of capillary hemangioma were described as well-marginated, regularly shaped, T2-hyperintense, iso- or hyperintense on T1-weighted images, and homogeneously enhanced with gadolinium [3, 6]. In our cases, the first case showed T2- and T1-isointense and contrast-enhanced mass, and the second case showed T2- and T1-hypointense mass. This discrepancy could make radiological diagnosis more difficult. Myxopapillary ependymoma is especially difficult to discriminate from capillary hemangioma because they share similar imaging features and locations [6, 15]. Capillary hemangioma should be considered a differential diagnosis when a well-demarcated, homogeneously enhanced mass is identified in the lumbar region, especially accompanied by spinal dysraphism.

On the other hand, the diagnosis of infantile capillary hemangioma is confirmed by histopathologic findings. The typical histopathologic feature is the proliferation of capillary vessels with endothelial cells [2–4, 6–8, 10]. GLUT1 is known to be expressed in the endothelium of hemangioma [16]. Immunohistochemical staining for GLUT1 is used to distinguish hemangioma from vascular malformations. CD31, known as platelet endothelial cell adhesion molecule 1 (PECAM-1), is a transmembrane glycoprotein expressed by endothelial cells [17–19]. GFAP is the hallmark of glial cells in the central nervous system [20], and its positivity supports that our biopsied tissue contains RMC.

## Conclusion

Pediatric intramedullary spinal capillary hemangioma associated with spinal dysraphism is rare. Capillary hemangioma occurring in the lumbar region co-presenting with spinal dysraphism suggests a possible origin from pluripotent cells involved in the process of secondary neurulation. Capillary hemangioma should be considered a differential diagnosis when a well-demarcated mass with homogeneously strong enhancement is identified at the lumbar level on MRI.

## Data Availability

The data is available from the corresponding author upon reasonable request.
